# Risk factors and prediction models for bronchitis obliterans after severe Mycoplasma pneumoniae pneumonia with plastic bronchitis in children

**DOI:** 10.12669/pjms.42.3.13709

**Published:** 2026-03

**Authors:** Fang Cheng, Ruixue Sun, Yingmian Zhao, Shixin Sun, Yunfeng Ma, Shuqing Feng

**Affiliations:** 1 Fang Cheng, Department of Pediatrics, Xingtai People’s Hospital, Xingtai Emergency Center, Xingtai 054000, Hebei, China; 2 Ruixue Sun, Department of Pediatrics, Xingtai People’s Hospital, Xingtai Emergency Center, Xingtai 054000, Hebei, China; 3 Yingmian Zhao, Department of Pediatrics, Xingtai People’s Hospital, Xingtai Emergency Center, Xingtai 054000, Hebei, China; 4 Shixin Sun, Department of Pediatrics, Xingtai People’s Hospital, Xingtai Emergency Center, Xingtai 054000, Hebei, China; 5 Yunfeng Ma, Department of Pediatrics, Xingtai People’s Hospital, Xingtai Emergency Center, Xingtai 054000, Hebei, China; 6 Shuqing Feng, Department of Pediatrics, Xingtai People’s Hospital, Xingtai Emergency Center, Xingtai 054000, Hebei, China

**Keywords:** Bronchitis obliterans, Bronchiolitis obliterans, Electronic bronchoscopy, Plastic bronchitis, Nomogram model, Prediction, Severe Mycoplasma pneumoniae pneumonia

## Abstract

**Objective:**

To investigate the relevant risk factors for bronchitis obliterans and develop a predictive nomogram for bronchitis obliterans in children with severe Mycoplasma pneumoniae pneumonia(SMPP).

**Methodology:**

This was a retrospective study. This study analyzed the clinical data of Xingtai People’s Hospital, Xingtai Emergency Center children with SMPP from September 2021 to September 2025, and categorized them into the bronchitis obliterans and PB groups. SPSS 26.0 statistical software was used for data organization and analysis.

**Results:**

Four hundred and sixty-four children with SMPP were finally included, of which 27(5.8%) children with PB were complicated by bronchitis obliterans. According to the chi square test and Mann Whitney U test, it was found that the gender, age, duration of fever, duration of cough, white blood cells, neutrophils, eosinophils CRP, alanine aminotransferase IL-6, IL-10, the comparison of tumor necrosis factor, APTT, D-dimer, aspartate aminotransferase, immunoglobulin E, and immunoglobulin G showed no statistically significant differences(P>0.05). The lactate dehydrogenase levels in patients with bronchitis obliterans were higher than those in patients with PB(P<0.05). The immunoglobulin A levels in patients with bronchitis obliterans were lower than those in patients with(P<0.05). The results showed that a higher level of LDH was an independent risk factor for bronchitis obliterans, while a higher level of immunoglobulin A was an independent protective factor for bronchitis obliterans.

**Conclusion:**

The increase of lactate dehydrogenase is an independent risk factor for bronchitis obliterans. The lactate dehydrogenase and immunoglobulin A have good sensitivity and specificity for building a nomogram prediction model for the risk of bronchitis obliterans.

## INTRODUCTION

Mycoplasma pneumonia (MP) is one of the common pathogens that cause community-acquired pneumonia (CAP) in children and adolescents.^1^ Previously, Mycoplasma pneumoniae pneumonia(MPP) was considered as a benign self-limiting disease. However, the incidence of RMPP complications is relatively high, even including necrotizing pneumonia (NP), bronchitis obliterans, bronchitis obliterans (BO) or thrombosis.^2^

Bronchitis obliterans is a long-term community-acquired pneumonia in children, the pathogenesis may be infection causing damage to the airway mucosa injury and induction of strong inflammatory response in the body, granulation tissue, and neovascularization.^3^ Previously, bronchitis obliterans was diagnosed through lung tissue biopsy or histopathological examination.^4^ With the development of bronchoscopy technology in children, fibrous branches tracheoscopy has gradually become the main method for diagnosing and treating bronchitis obliterans.^5^ Bronchitis obliterans and occlusive disease different from bronchiolitis obliterans, the main pathological manifestation is small and medium-sized bronchioles containing cartilage.

The proliferation and fibrosis of inflammatory granulation tissue in the trachea can block the bronchial lumen chronic airflow obstruction syndrome, clinically characterized by atelectasis or pulmonary hypoplasia leaf collapse is the main manifestation,^5^ and sub segments can be seen under electronic bronchoscopy distal bronchial occlusion in the subsegmental segment, with some cases accompanied by proximal bronchial obstruction dilation of the lumen, atrophy of the mucosa, and exposure of the cartilage ring.^6^ Therefore, it is quite essential to find a feasible and accurate screening tool to identify the bronchitis obliterans high-risk population of RMPP patients, which will help the early intervention of bronchitis obliterans.

The nomogram is a popular prognostic tool that can predict clinical events by integrating potential risk factors.^7^ The nomogram has been widely used in tumor prognosis and supports the development of personalized oncology medicine.^8^ Recently, a nomogram has been effectively used to predict the short-term and long-term survival rates of asymptomatic adults screened for cardiac risk factors.^9^ Moreover, a nomogram is also used to predict the risk stratification of diseases, including myocardial infarction and stroke.^10^ In the current study, we retrospectively analyzed the clinical characteristics, risk factors, and the application and timing of corresponding treatment measures in children with RMPP and bronchitis obliterans. Importantly, we established a nomogram prediction model to predict the risk of bronchitis obliterans in children with SRMPP, with the hope to provide ideas for the early diagnosis of bronchitis obliterans and the timing of intervention.

## METHODOLOGY

This was a retrospective study. This study included SMPP patients (1-14 years old) who were diagnosed in the Department of Pediatrics, Xingtai People’s Hospital, Xingtai Emergency Center from September 2021 to September 2025. SMPP was defined as:

- sputum culture, serum etiology and lung CT suggested mycoplasma pneumoniae pneumonia in children;
- a sustained fever for seven days or more;
- pneumonia caused by Mycoplasma pneumoniae (MP) infection, with the condition meeting the diagnostic criteria for severe community-acquired pneumonia(CAP).

### Ethical approval:

The study was approved by the Institutional Ethics Committee of Xingtai People’s Hospital (No.:2025[015]; Date: February 13, 2025), and written informed consent was obtained from all participants’ guardians.

### Inclusion criteria:

- Meet the diagnostic criteria for community-acquired pneumonia in children.^11^
- The fiberoptic bronchoscopy was performed in the hospital, and tracheobronchial occlusion was found under the bronchoscopy.
- For the PB group, the following criteria were met: (1) Children with SMPP were diagnosed; (2) There was no bronchitis obliterans-related manifestation under bronchoscope.^12^

### Exclusion criteria:

- Patients with tuberculosis, asthma, repeated respiratory infections, bronchiectasis, or other chronic lung diseases.
- With a history of severe pneumonia before.
- With a history of congenital heart disease, chronic liver and kidney insufficiency, and immunodeficiency.

### Data collection:

The clinical information was collected retrospectively from the patients’ medical records during acute infection, including age, gender, clinical symptoms and signs, intrapulmonary and extrapulmonary complications, fever duration and treatment. All patients underwent chest X-ray examination on admission, showing clear focal or segmental infiltration, with or without pleural effusion, and whether the consolidation area exceeded 2/3 of the lung lobes. Peripheral blood samples were collected on admission to determine complete blood count, albumin (ALB), C-reactive protein (CRP), PCT, lactate dehydrogenase (LDH), and T lymphocyte subsets. In addition to the MP-RNA test and MP-IgM test, other microbiological tests were carried out, including protein purification derivative tests, blood bacterial cultures, BALF bacterial cultures, and nasopharyngeal aspirates/swabs to detect common respiratory virus antigens (respiratory syncytial virus, influenza virus, adenovirus and parainfluenza virus), and serological testing of chlamydia pneumoniae.

### Statistical analysis:

Data were collated and analyzed using SPSS 26.0 statistical software. The confidence intervals is 95%. For continuous variables, if the data did not conform to a distribution, the median(interquartile range) was used for description, and the Mann-Whitney U test was used for comparison between groups. C variables were expressed as frequencies(percentages), and the chi-square test was used for comparison between groups. Multivariate analysis was performed using binary logistic regression analysis. The prediction performance was analyzed using ROC curve analysis. P< 0.05 was considered statistically significant.

## RESULTS

A total 464 of patients with confirmed PB were included in this study. Among the 464 cases of PB patients, Outcome of Obstructive bronchitis occurred in 27 patients with PB. All patients had symptoms and signs of pneumonia, including fever, cough, and abnormal breath sounds on auscultation. There was no significant difference in gender duration of fever, duration of cough, white blood cell, neutrophil, eosinophil, CRP, alanine aminotransferase, IL-6, IL-10, tumor necrosis factor, PTT, D-dimer, aspartate aminotransferase, immunoglobulin E, immunoglobulin G and age at diagnosis between the PB group and the bronchitis obliterans group (Table-I). However, the lactate dehydrogenase level was higher in the bronchitis obliterans group than in the plastic bronchitis group (P < 0.05). The immunoglobulin A was lower in the obstructive bronchitis group than in the plastic bronitis group (P < 0.05).

**Table-I:** Univariate analysis of the plastic bronchitis group and the bronchitis obliterans group

Clinical index	PB(n=437)	bronchitis obliterans(n=27)	χ^2^/z	P
Gender, n(%)			2.788	0.095
Male	219(50.1)	18(66.7)		
Female	218(49.9)	9(33.3)		
Age (years old)	7.00(5.00, 9.00)	8.00(5.00, 9.00)	-0.833	0.405
Fever time (days)	6.00(4.00, 7.00)	5.00(4.00, 7.00)	-1.612	0.107
Cough time (days)	7.00(4.00, 10.00)	7.00(4.00, 17.00)	-0.582	0.561
WBC count(1 × 10^9^/L)	8.71(6.61, 12.48)	7.38(4.42, 10.81)	-1.742	0.082
Neutrophil (%)	5.21(3.65, 7.92)	5.02(2.84, 8.05)	-1.135	0.256
Eosinophil(%)	0.07(0.01, 0.21)	0.04(0, 0.16)	-1.347	0.178
CRP (mg/L)	7.06(0.59, 21.23)	11.00(2.30, 42.01)	-1.525	0.127
ALT	15.20(11.20, 25.80)	19.10(14.80, 24.00)	-1.475	0.140
LDH	341.95(280.80, 423.13)	460.40(364.30, 667.10)	-3.211	0.001
IL-6	34.83(15.68, 83.47)	37.44(6.00, 58.96)	-0.802	0.422
IL-10	9.11(5.67, 15.00)	7.88(5.42, 11.47)	-1.060	0.289
TNF	7.13(4.19, 12.63)	9.78(5.09, 14.99)	-1.313	0.189
APTT	31.60(27.50, 35.00)	30.90(26.00, 35.60)	-0.256	0.798
D-dimer	0.49(0.25, 1.31)	0.69(0.27, 2.42)	-1.104	0.270
AST	30.35(24.53, 38.80)	34.40(27.20, 44.10)	-1.870	0.062
Ig E	76.00(31.00, 200.00)	66.00(23.00, 120.00)	-0.948	0.343
Ig G	8.76(7.12, 10.33)	8.80(7.07, 11.01)	-0.365	0.715
Ig A	1.48(0.98, 2.01)	1.17(0.85, 1.47)	-2.303	0.021
Erythra, n(%)	3(0.7)	0(0)	-	1.000

Taking the group status of plastic bronchitis group and bronchitis obliterans group as the dependent variable (shaping bronchitis obliterans group value =1, value of plastic bronchitis group =0), the univariate analysis indicators between plastic bronchitis group and bronchitis obliterans group were different, including LDH and immunoglobulin A as independent variables, and the method selected “input”. The results found that higher LDH levels were an independent risk factor for bronchitis obliterans, while higher immunoglobulin A levels were an independent protective factor affecting bronchitis obliterans. Based on the results of the binary logistic regression analysis, the equation of the logistic regression model was logit(P)=-2.604+0.002 LDH-0.895 immunoglobulin A (Table-II).

**Table-II:** Multivariate regression analysis of the plastic bronchitis group and the bronchitis obliterans group.

Clinical index	β	SE	Wald χ^2^	P	OR	95%CI	
						Lower limit	Upper limit
LDH	0.002	0.001	5.111	0.024	1.002	1.000	1.003
IgA	-0.895	0.422	4.495	0.034	0.408	0.179	0.935
Quantity	-2.604	0.607	18.407	<0.001	0.074		

**Fig.1:**
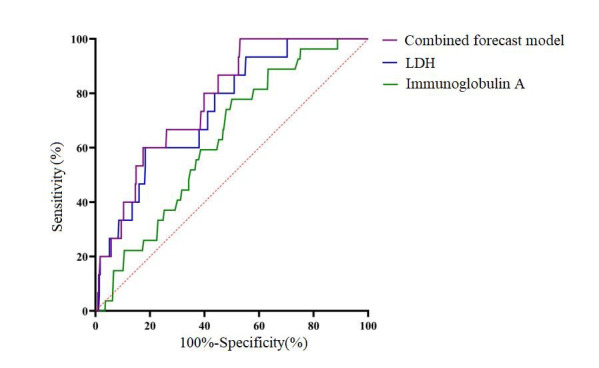
ROC plot of LDH and immunoglobulin A and their combination on bronchitis obliterans group.

ROC curve analysis included LDH and immunoglobulin A as the status variables (Obliterative bronchitis group assignment value=1 and plastic bronchitis group assignment value=0). We found that the AUC area of “LDH + immunoglobulin A” was 0.780 (0.683,0.876) (P <0.001), with a specificity of 0.470, a sensitivity of 1.000 and a Youden index of 0.470. The AUC area of LDH for predicting bronchitis obliterans was 0.744 (0.630~0.858) (P=0.001), specificity of 0.817, sensitivity of 0.600, Yoden index of 0.417 and optimal cutoff value of 458.45. The AUC area of immunoglobulin A for predicting bronchitis obliterans was 0.674 (0.581~0.768) (P=0.022), with specificity of 0.516, sensitivity of 0.933, and Yoden index of 0.449 with an optimal cutoff value of 1.475. Table-III and Fig.1.

**Table-III:** Analysis of risk prediction efficacy in plastic bronchitis and bronchitis obliterans groups.

Index	Cutoff value	AUC	95%CI		P	Specificity	Sensitivity	Yoden index
			Lower limit	Upper limit				
Joint prediction model	-	0.780	0.683	0.876	<0.001	0.470	1.000	0.470
LDH	458.45	0.744	0.630	0.858	0.001	0.817	0.600	0.417
IgA	1.475	0.674	0.581	0.768	0.022	0.516	0.933	0.449

## DISCUSSION

In this study, we identified 20 associated factors for risk of childhood plastic bronchitis based on univariate and multivariate regression analysis, including leukocyte count, neutrophil proportion, eosinophil proportion, immunoglobulin level, cytokine level, alanine aminase, D dimer, all used to establish a nomogram of risk of bronchitis obliterans. To our knowledge, this is the first nomogram study of the risk contrast made by plastic bronchitis versus bronchitis obliterans. This new nomogram showed satisfactory performance in the study cohort based on the AUC and calibration curve evaluation. Therefore, this nomogram can be effectively applied in clinical practice and can help to predict the long-term development of bronchitis obliterans in children due to SMPP. Previous studies used multivariable regression analysis to determine the risk of extrapulmonary complications such as pulmonary necrosis^13^ and endobronchial mucus embolization^14^ in patients with MPP. However, the research on the risk of plastic bronchitis in SMPP patients with bronchitis obliterans has not been reported. Bronchitis obliterans and bronchiolitis obliterans is different, and the main pathology is the small and medium branches containing cartilage The proliferation and fibrosis of the tracheal inflammatory granulation tissue blocks the bronchial lumen And produce chronic airflow obstruction syndrome, clinically to atelectasis or lung Leaf collapse was the main manifestation of, subsegments and electronic bronchoscopy Subsubdistal bronchial occlusion, in some cases with proximal bronchus Luminal expansion, mucosal atrophy, and cartilage ring were exposed to.^15^ The mechanism of forming obstructive bronchitis after mycoplasma infection is still known undefined, and may have both inflammatory and immune responses involved.^16^

LDH is a cytoplasmic enzyme widely distributed across major organ systems. When cell lysis occurs or cell membranes are damaged, LDH is released into the extracellular space.^17^ LDH, a cytoplasmic enzyme present in multiple vital organs, serves as a nonspecific inflammatory biomarker reflecting tissue lysis or cellular membrane damage, and is clinically utilized to monitor infection severity.^18^ Previous studies have demonstrated that LDH levels rise significantly following MP infection and exhibit a positive correlation with disease severity^19^ and have suggested that LDH is an early indicator for the initiation of glucocorticoid therapy, with effective ranges from 302 to 480 U/L.^20^ Our study identified markedly elevated LDH levels in the bronchitis obliterans group, further establishing LDH as an independent risk factor for bronchitis obliterans development in children with SMPP.

The relationship between bronchiotis obliterans and immunoglobulin A(IgA) involves multiple links such as immune-mediated inflammatory response, mucosal barrier disruption, and abnormal fibrosis repair. There is currently limited direct research on bronchiotis obliterans and IgA, based on their pathogenesis and the biological functions of IgA, the following associations can be inferred:-Mycoplasma pneumonia(MP) is one of the important causes of childhood obstructive pulmonary disease, and MP infection can affect IgA levels. Mycoplasma pneumonia (MP)infection can affect IgA levels. The immunoglobulin A levels in patients with obstructive bronchitis are lower than those in patients with obstructive bronchitis for unknown reasons.

ROC curve analysis was performed using LDH and immunoglobulin A as test variables, with the grouping of the PB group and the bronchitis obliterans group as state variables (assigned a value of one for bronchitis obliterans group and 0 for PB group). The results showed that the combined prediction of “LDH+immunoglobulin A” had an AUC area of 0.780 (0.683, 0.876)(P<0.001), specificity of 0.470, sensitivity of 1.000, and Youden index of 0.470 for predicting obstructive bronchitis. The AUC area of LDH for predicting bronchitis obstructive is 0.744(0.630~0.858) (P=0.001), specificity is 0.817, sensitivity is 0.600, Youden index is 0.417, and the optimal cutoff value is 458.45. Immunoglobulin A has an AUC area of 0.674 (0.581~0.768)(P=0.022), specificity of 0.516, sensitivity of 0.933, Youden index of 0.449, and optimal cutoff value of 1.475 for predicting obstructive bronchitis.

### Limitations:

Although our nomogram performed well in both the development and validation dataset, there are several limitations to this study. First, the nomogram was developed and validated using single-center data, which may restrict the generalizability of the model to other populations or regions. Second, patients with pre-existing conditions such as asthma, chronic respiratory diseases, or immune-related disorders were excluded to minimize confounding factors.

## CONCLUSIONS

The incidence of plastic bronchitis after severe mycoplasma pneumonia in children is high, among which the incidence of bronchitis obliterans is high and serious. The increase of lactate dehydrogenase is an independent risk factor for bronchitis obliterans. The lactate dehydrogenase and immunoglobulin A have good sensitivity and specificity for building a nomogram prediction model for the risk of bronchitis obliterans.

### Authors’ Contributions:

**FC** and **RS:** Participated in data analysis and prepared the original manuscript, is responsible and accountable for the accuracy or integrity of the work.

**YZ** and **SS:** Designed the study and reviewed manuscript.

**YM** and **SF:** Screened the eligible study participants.

All authors have read and approved the final manuscript.
